# Supplementary figures and images for: The Xanthine Oxidase Inhibitor Febuxostat Suppresses Adipogenesis and Activates Nrf2
Source: Antioxidants (Basel). 2023 Jan 5;12(1):133. doi: 10.3390/antiox12010133 (PMC9854541; doi:10.3390/antiox12010133)

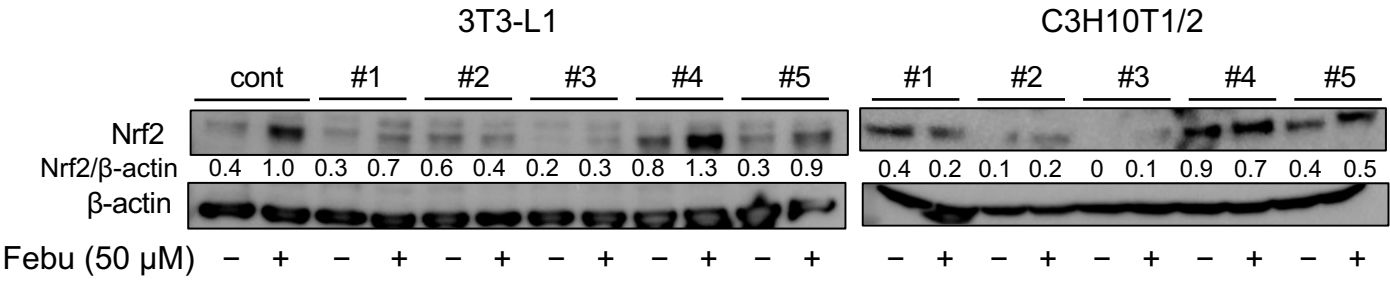

Figure S1

Supplement: Supplementary file 1 [file antioxidants-12-00133-s001.zip › antioxidants-2141382-supplementary.pdf]
